# General practitioners attitude and confidence scale for dementia (GPACS-D): confirmatory factor analysis and comparative subscale scores among GPs and supervisors

**DOI:** 10.1186/s12875-018-0896-1

**Published:** 2019-01-08

**Authors:** Ron Mason, Kathleen Doherty, Claire Eccleston, Michael Annear, Amanda Lo, Laura Tierney, Fran McInerney, Andrew Robinson

**Affiliations:** 1 Wicking Dementia Research and Education Centre, College of Health and Medicine, University of Tasmania Private Bag 143, Hobart, Tasmania 7001 Australia; 2 School of Medicine, College of Health and Medicine, University of Tasmania Private Bag 34, Hobart, Tasmania 7001 Australia; 3 Department of Health and Physical Education, International Christian University, 3-10-2 Osawa, Mitaka City, Tokyo 181-001 Japan

**Keywords:** Attitudes, Confidence, Dementia education, General practitioner, Scale validation

## Abstract

**Background:**

The attitude of General Practitioner’s (GP’s) towards dementia and confidence in their clinical abilities impacts on diagnosis rates and management of the condition. The purpose of the present research is to refine and confirm the reliability and validity of the General Practitioner Attitudes and Confidence Scale for Dementia (GPACS-D) as a tool to measure confidence and attitude.

**Methods:**

A sample of 194 GP volunteers attending dementia education workshops were recruited to complete the GPACS-D before and after the workshop. Volunteer respondents comprised both GP Registrars and GP Supervisors. Analyses included Confirmatory Factor Analysis (CFA), measures of internal consistency, Pearson correlations, and a comparison of subscale scores between cohorts (T-Test for independent samples).

**Results:**

Findings of the CFA support a 15-item, 3-factor model with four items removed due to poor performance and one item moved between factors. The resultant model exhibited good fit (x^2^ = 103.88; *p* = .105; RMSEA = .032; PCLOSE = .915; CFI = .967; TLI = 960), with acceptable internal consistency. Subscales exhibited clear discriminant validity with no underlying relationships between subscales. Finally, total and subscale scores exhibited good discrimination between groups who would be expected to score differently based on experience and level of exposure to dementia.

**Conclusion:**

The 15-item, 3-subscale GPACS-D is a reliable and valid measure of GP confidence and attitudes toward dementia. The subscales clearly distinguish between groups who might be expected to score differently from each other based on their training or professional experiences. The psychometric properties of the GPACS-D support its use as a research tool.

## Background

Timely and accurate diagnosis of dementia is widely acknowledged to be beneficial to health care providers, patients and their families through more effective management of symptoms and concerns, prompt care planning and a proactive approach to social support [1]. However, low rates of diagnosis persist, with a recent study determining that 59% of adults with probable dementia have either not been diagnosed or are unaware of their diagnosis [2]. This has been attributed to multiple factors involving the health care provider, the patient, their families and the health system [3]. For the family physician, their attitude toward dementia, including the potentially stigmatising impact of a dementia diagnosis and concerns about its benefits, together with difficulty in communicating effectively with patients and their families have been identified as important contributing factors to low diagnosis rates [3]. While GP attitudes toward caring for people with dementia have been shown to be positive [4], fear of misdiagnosis and lack of confidence in diagnostic and dementia management skills have been reported to be of particular concern in multiple studies with a lack of effective education and training frequently cited as an underlying cause [5–7].

Intervention trials of dementia education and training programs often use rates of detection, and compliance with practice guidelines as key outcome measures [8, 9], however assessment of practitioner confidence and attitudes are equally important indicators of educational effectiveness [8]. Where this has been explored, dementia education can be shown to improve practice quality while having little positive impact on the knowledge or attitude to dementia of primary care providers [10]. Survey based measures have been used to establish attitudes and/or confidence levels of health practitioners toward dementia [7, 11], or in depth interviews used to explore attitudes and barriers to diagnosis [12]. O’Connor and McFadden [13] reported the development of the Dementia Attitudes Scale which has been used to explore attitudes to dementia in medical students and other groups of health care professionals, but this tool does not address issues associated with confidence which have particular relevance to general practice. Liu and co-workers (2013) used a postal questionnaire to compare attitudes to dementia of physicians who had or had not received dementia specific training. Using exploratory factor analysis (EFA) two factors were identified: confidence and negative views, which underpinned attitudes to dementia. Those who had received training were more confident and held less negative views, although the type of training received was not controlled in this study as it was self-reported [14].

Given that physicians report the need for better education about dementia as a step toward practice change and acknowledging the importance of confidence and attitude towards dementia in effecting change, evaluation of the effectiveness of educational interventions should include assessment of both confidence and attitude. In a recently published paper [15], the authors reported the development of the General Practitioner Attitudes and Confidence Scale for Dementia (GPACS-D). Informed by social psychological theories of health and practice behaviour [16], principal component analysis (PCA) resulted in four hypothesised subscales; *Confidence in Clinical Abilities; Attitude to Care; Attitude to Communication* and *Fears and Frustrations*. These subscales reflect an inter-relationship between attitudes, confidence (self-efficacy or perceived behavioural control), intention and resultant behaviours [3, 14].

*Confidence in Clinical Abilities* subscale includes items addressing diagnostic and clinical abilities [4], management of symptoms associated with the syndrome [14] and knowledge of external resources, including respite services [6, 17]. The *Attitude to Care* subscale focusses on attitudes to early diagnosis [3], the role of the GP in organising care, including external support [7], and the inclusion of both the patient and carer/s in the diagnosis and future management of dementia [11]. The *Fears and Frustrations* subscale reflects frustration with and/or avoidance of treating dementia, and includes difficulty in diagnosing dementia or the management of dementia related symptoms [5, 14] and a preference for treating other conditions. The *Attitude to Communication* subscale reflects perspectives on diagnosis disclosure to the patient so they can plan for the future [18], as well as informing patients and their families of the terminal nature of the condition [1]. The preliminary GPACS-D scale demonstrated sound psychometric properties, with each survey item exhibiting good test retest reliability, sensitivity to change and good internal consistency [15].

The purpose of the present research is to confirm the reliability and validity of GPACS-D as a tool to measure GP attitudes and confidence towards dementia. The construct validity of the hypothesised GPACS-D subscales were established by undertaking confirmatory factor analysis (CFA) to evaluate the adequacy of the model resulting in a final model suitable for research application.

## Methods

### Sample

Purposive sampling was employed to recruit GP supervisors and registrars (GPR’s) participating in Recognising, Diagnosing and Managing Dementia in General Practice Workshops conducted in three Australian states: Tasmania, New South Wales and Queensland between June and December 2016. GPRs are medical graduates undertaking a vocational training program to specialise in general practice (Australian Government Department of Health 2017). GP supervisors are experienced GPs who have regular contact with registrars and oversee their patient care, provide support and feedback to facilitate learning (Australian Government Department of Health 2017). Volunteer participants were provided with information about the research prior to their participation in the workshop and invited to complete the survey. Sample size adequacy was determined according to criteria set out by Tabachnick et al. (2001), where a subject to item ratio of 10:1 is desirable for CFA. Our data set comprised 194 cases and was therefore considered acceptable to draw inferences from the data [19]. Completion of the survey implied consent consistent with Australian National Health and Medical Research Council guidelines. A University Human Research Ethics Committee reviewed and approved this study (Reference Number: H0012046).

### Measure

The survey comprises 20 items designed to address GP attitudes towards (a) diagnosis and treatment, (b) confidence in clinical skills and (c) awareness of support networks for dementia. Survey items were measured via a 5-point Likert Scale (1 = strongly disagree, 5 = strongly agree). Because no a priori assumptions were made about the relationship amongst the variables in the original survey, Principal Component Analysis (PCA) was previously employed to reduce the set of observed variables to a smaller, more interpretable structure and to identify potential constructs for further examination and refinement [20, 21] prior to further validation. The present study sought to confirm these constructs as valid, reliable and independent subscales within the GPACS-D measure using CFA.

### Data analysis

All analyses were undertaken using SPSS (Version 22) and AMOS (for structural equation modelling). Because estimation procedures are dependent upon the distribution of the data, an analysis of data properties was undertaken to establish the extent to which data was normally distributed. Data were highly skewed and kurtotic, however each were within acceptable parameters. Curren (1996) suggests that univariate skewness > 2 and kurtosis > 7 present significant problems for maximum likelihood estimation. In our sample univariate skewness ranged from − 1.328 to 0.108, with a mean skewness of 0.192, while univariate kurtosis values range from − 0.747 to 0.081, with a mean kurtosis of 0.246. While it is necessary to check for univariate normality it is not always a sufficient condition for multivariate normality.

An important assumption is that data are *multivariate normal,* with Bentler (2005) suggesting that values equal to or greater than 5 represent departure from multivariate normality [22]. Our Critical value of 3.375 falls within acceptable limits. Given this maximum likelihood estimation was considered appropriate for model development.

Confirmatory Factor Analysis (CFA) was undertaken to assess the quality of the hypothesised factor structure [23] previously identified via principal components analysis (PCA) [15]. CFA also sought to confirm the construct and discriminant validity of each of the subscales. Parameter estimates were examined to establish utility, while potential item misspecification was identified through an examination of the standardised residuals (values > 1.96) and modification indices (values <.30) [24–26].

Consistent with CFA reporting conventions, goodness of fit measures included Chi Square, Root Mean Square Error of Approximation (RMSEA; values < 0.06 are desirable); (PCLOSE; values > 0.50 are desirable); Comparative Fit Index (CFI; values > 0.95 are desirable) and Tucker-Lewis Index (TLI; values >.95 are desirable). The reporting of multiple fit indices is common practice and is recommended when assessing model fit to support the reliability of the findings [26–31].

### Comparative analysis

Factor scores generated from the CFA were used to compute standardised summary scores for each subscale and a total score. Because of reverse scoring a higher score for *Fears and frustrations* indicate less frustration. These scores were used to measure differences between different cohorts based on experience and exposure to dementia. T-tests for independent samples were employed to establish potential differences between groups for total and subscale scores.

## Results

A total of 194 respondents completed the survey with a response rate of 93% (See Table 1). The sample comprised 39% supervisors (*n* = 76) and 61% GP Registrars (*n* = 118). The mean age of respondents was 37 years of age (*SD* = 8.70), 54% were female (*n* = 93) and 38% were born in Australia (*n* = 72). See Table 1 for demographic information.

**Table 1:** Demographic Information

	Sample Size (*n* = 194)
Mean Age	37.2 (SD = 8.70)
Age Range	25–66
Male Respondents	(*n* = 85) 45.7%
Australian Born	(n = 72) 38%
Occupational Groups	
Registrar	(n = 118) 61%
	*Male* (44%)
Supervisor	(*n* = 76) 39%
	*Male* (56%)
Prior Dementia Education	(*n* = 18) 9%
Family member with dementia	(*n* = 68) 36.6%
Provided Professional Services for person with dementia	(*n* = 162) 84%

### Confirmatory factor analysis

The initial 20-item four factor model hypothesised by the PCA returned a significant Chi Square statistic (x^2^ = 247.62; *p* = .000) indicating a lack of fit between the hypothesised model and the observed data. Post-hoc analyses were undertaken to refine the model as a result of the initial CFA [23, 32], which resulted in 5 items being removed from the original 20 item model. Two items were eliminated because of non-significant loadings on their respective constructs; Item 11, *The term dementia should be avoided when discussing a diagnosis with a carer/family member,* and item 16, *Dementia is better treated by specialist physicians.* An examination of modification indices revealed Item 12, *Patients with dementia should be informed early so they can plan for the future,* cross loading with a number of items, especially those reflecting the *Attitude to Care subscale.* As a result, Item 12 was moved to improve factor interpretability and model fit [26, 32]. While the initial decision to move item 12 was based on statistical criteria, an examination of the item also suggested conceptual congruity with *Attitude to Care* because it addressed perceptions of the benefits of early diagnosis and future care outcomes.

The construct *Attitude to Communication* was removed from the analysis because of the elimination of Item 11 (non-significant loading) as well as potential redundancy between the 2 remaining items (Item 13, *It is important to inform the person of the terminal course of the condition, and* Item 14, *It is important to inform relatives/family carers of the terminal course of the condition*. Bivariate correlations indicated redundancy (*r* = .760) and an examination of the items confirmed this. Both items related to the importance of informing the patient and family of the terminal course of the condition, with the only difference between the items being the subject. Finally, an examination of the standardised residuals identified potential misspecification of Item 9, *Guidelines for the management of dementia would greatly assist in providing care.* Examination of the item suggested that it reflected a desire for guidelines rather than an attitude to care. Removal of the item resulted in a significantly improved overall model fit. After the removal of redundant, cross-loading and mis-specified items, and improvements in factorability of the subscales a final 15 item, 3-factor model was confirmed.

**Fig. 1:**
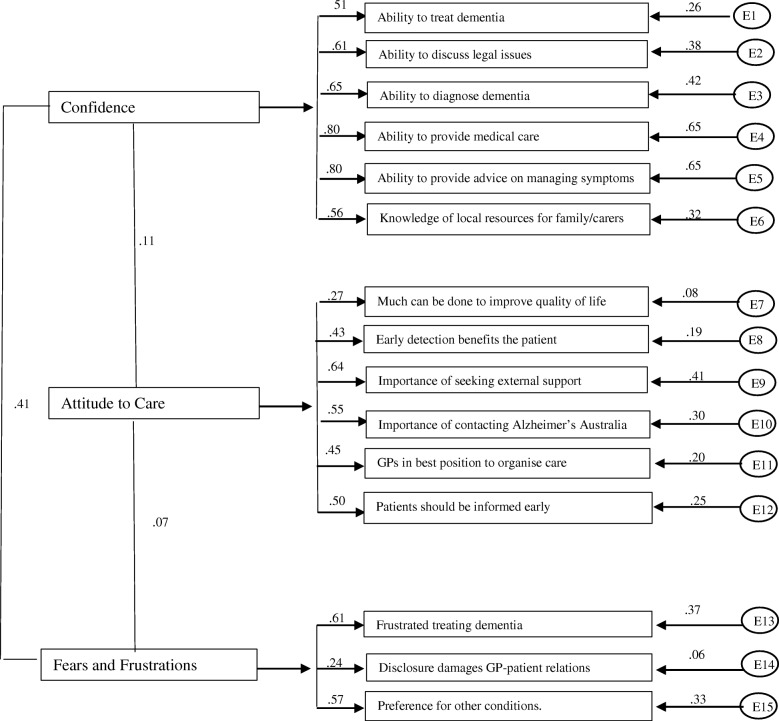
Confirmation of three factor, 15 item model for GPACS-D

The final 15-item, three-factor model (Fig. 1) exhibited very good fit, confirming construct validity of the revised model, (x^2^ = 103.88; *p* = .105; RMSEA = .032; PCLOSE = .915; CFI = .967; TLI = .960). All items were significantly correlated to the construct, and modification indices were acceptable (*r* < .30), indicating no underlying significant relationships between items or constructs. Inter correlations between constructs were acceptable with coefficients below the .50 criteria [31]. The correlations between *Attitude to Care* and *Confidence in Clinical Abilities* was .11; *Attitude to Care* and *Fears and Frustrations* (.07); and *Confidence in Clinical Abilities* and *Fears and Frustrations* (.41). These results indicate minimal underlying correlations between constructs and clear discriminant validity.

### Internal consistency

Total score for overall scale score (α = .765) indicated acceptable internal consistency for a 15-item GPACS-D. Cronbach’s alpha scores for two constructs reflected adequate internal consistency: *Confidence in Clinical Abilities* (a = .810); and *Attitude to Care* (a = .769). *Fears and Frustrations* (a = .450) exhibited a comparatively low internal consistency score. However, given the construct was defined by only three items, and because Cronbach’s alpha is affected by the number of items that define a construct or latent variable, this may account for the low score and underestimate reliability [33].

### Differences between groups

A comparative analysis was undertaken to determine whether the hypothesised sub scales could detect differences between different groups of GPs on the basis of exposure to dementia or experience as a GP. It was hypothesised that certain cohorts, because of their experience, would score higher than those with less experience or exposure to dementia.

As shown in Table 2, GP Supervisors were more confident in their clinical abilities than GP Registrars (f = 1.48; *t* = .283; *p* < .000), with similar results emerging for the fears and frustrations subscale (f = .447; *t* = 4.72; *p* < .000). Additionally, those with prior professional experience of treating someone with dementia (irrespective of professional title) recorded a higher score for confidence in clinical abilities (f = .332; *t* = 4.26; *p* < .000) and fears and frustrations (f = .426; *t* = 2.69; *p* < .008) than those who had not. No significant differences emerged for attitude to care. The overall summative score (combined total scores for confidence, attitude to care and fears and frustrations) reflected these differences, with GP Supervisors and those who had professional experience with clients with dementia scoring significantly higher than GP Registrars (f = .396; *t* = 6.26; p < .000), and those who had not treated clients with dementia in a professional capacity (f = .05; *t* = 4.47; *p* < .000).

**Table 2:** Total and subscale scores (standardised) by Role and Experience

		Subscale mean scores/sd/total		
	Total mean score/sd/15	Confidence in clinical abilities/sd/5	Attitude to Care/sd/5	Fears and Frustrations/sd/5^b^
Alpha (a)	a = .765	a = .810	a = .765	a = .450
Role				
GP Registrars (*n* = 118)	9.96/1.21	2.66/.63	4.32/.45	2.98/.75
Supervisors (*n* = 76)	11.1/1.30^a^	3.28/.76^a^	4.34/.41	3.49/.69^a^
Provided Professional Dementia Care				
Yes (*n* = 162)	10.59/1.30^a^	2.99/.70^a^	4.36/.41	3.24/.77^a^
No (*n* = 28)	9.41/1.26	2.37/.78	4.21/.52	2.83/.71

^a^indicates a statistically significant difference at the .001 level of significance. T Test for independent samples

^b^Because of reverse scoring a higher score indicates less fear and frustration

These findings suggest that the GPACS-D can differentiate between groups based on their level of exposure to, or experience of dementia, either from providing professional services, or being more experienced as a GP.

## Discussion

The GPACS-D was developed over two distinct phases. Phase one involved the development and pilot testing of the GPACS-D and subsequent PCA to establish the preliminary validity of the scale and hypothesised subscales [15]. In phase two, described in this paper, we undertook a CFA to refine and finalise the scale as well as establish the construct and discriminant validity of each sub scale and the items reflecting them.

The final and validated GPACS-D survey comprises 15 items and 3 subscales; *Confidence in Clinical Abilities (6 items; a = .869)*, *Attitude to Care* (6 items; a = .765) and *Fears and Frustrations (*3 items; a = .450*).* Each of the subscales confirmed in the model identifies specific aspects of attitude, confidence and frustrations implicated in the diagnosis and treatment of dementia. Both summative and subscale scores identified significant differences between groups, confirming the construct validity of the 15 item GPACS-D. Of note, supervisors and those who had provided professional service to people with dementia scored higher on all 3 subscales demonstrating that the scale is sensitive and applicable to analysis of differences in attitude to dementia care, confidence in diagnostic and clinical skills and frustration associated with treating dementia.

Our final model retained three of the four original constructs identified through PCA. *Attitude to Communication* was eliminated during the CFA modelling process due to redundancy and limited scope of the two remaining items. Communication has been identified as a key determinant in missed and delayed diagnosis and as an obstacle to effective doctor-patient communication about dementia [11]. Communication about dementia is complex and involves several players (GPs, people living with dementia, family members and other health care providers) and multiple issues ranging from difficulties in communicating a diagnosis, to poor literacy skills and cultural considerations [3]. It is likely that a more extensive, potentially stand-alone tool with a larger number of items would better address the complexity of these issues and identify elements of communication that can be targeted to improve communication between the physician, family, carers and the person living with dementia.

The *Attitude to Care* and *Fears and Frustrations* subscales were independent of each other while the *Fears and Frustrations* subscale was moderately inversely related to *Confidence in Clinical Abilities*. Several surveys have been undertaken to investigate attitude and confidence of GPs in other areas of health care such as diabetes [34], drug use [35], smoking cessation [36] and health promotion [37]. Negative beliefs and lack of confidence when discussing unpleasant or time-consuming topics were reported [36] as were avoidance of more difficult discussions [35]. These studies emphasise how confidence and attitudes impact on GP approaches to health-related issues and that changing clinical practice is not simply a matter of increasing knowledge but also addressing how a GP’s perceptions and beliefs affect their practice. Confidence in one’s ability to undertake an action is also recognised as an important predictor of behaviour [16, 38], and has been identified as a barrier to diagnosing and treating dementia [4]. Poor rates of diagnosis have been attributed to negative attitudes towards early diagnosis [12] and a reluctance to disclose [39, 40].

Regardless of attitudes towards the early diagnosis of dementia, a lack of confidence in clinical and management abilities may lead to avoidance of and delays in making a diagnosis. As with many people, health professionals tend to avoid those activities/behaviours for which they feel ill-equipped or exceed their capacity [9, 16], and within the context of dementia diagnosis and management, fears of professional inadequacy may contribute to frustration or avoidance of the condition resulting in low rates of diagnosis [7, 41, 42]. Avoidance of dementia may not be explicit but manifest in a reluctance to formalise a diagnosis, the preferential treatment of conditions for which treatment options are available, giving low priority to dementia symptoms compared to other health problems or avoiding care via the use of referrals because of a perception of having little to offer by way of treatment or cure [43]. Given that two of the items comprising the *Fears and Frustrations* construct align with avoidance, the construct may be more appropriately entitled ‘avoidance’ for the final version of the GPACS-D.

Improved education and training is often cited as the solution to poor diagnosis rates and management of dementia. However, the focus on knowledge and skills rather than behaviour and attitudes has been noted as a key gap in preparation for practice [44, 45]. As noted in other areas of health care, educational interventions need to address more abstract concepts such as nihilism, stigma and ageism as well as deficits in communication, disclosure and management skills [46].

Three of the four major factors identified by Bradford (2010) are covered by this tool including concepts relating to educational needs, attitudes towards dementia and approach to testing. All have been identified as factors contributing to either missed or delayed diagnosis of dementia [3].

The GPACS-D is a tool suitable to measure GP confidence and attitudes to dementia, which underpin behaviour change but are infrequently used as outcome measures in the evaluation of dementia educational outcomes [3].

### Limitations

*Attitude to Care* was negatively skewed as evidenced by the high mean score. This suggests that the participating GPs may have a pre-existing and favourable attitude towards engaging with dementia patients or that there is a form of social desirability bias at play [13]. As previous research has suggested, the impact of social norms in this group may account for self-reported favourable attitudes [13]. This point needs to be taken into consideration with self-administered surveys.

The internal reliability score for Fears and Frustrations was low and is partly a consequence of the small number of items that make up the subscale. The construct is relevant because it identifies concepts that arise as a response to managing dementia and is moderately associated with confidence. With respect to the communication subscale, the number of items left after model fitting was too low and the scope too limited to retain this subscale and should be addressed independently of this tool.

## Conclusion

The 15-item, 3-subscale GPACS-D is a reliable and valid measure of GP confidence and attitudes towards dementia.

## Abbreviations

CFA: Confirmatory Factor Analysis
GP: General Practitioner
GPACS-D: General Practitioners Attitude and Confidence Scale - Dementia
GPR: General Practitioner Registrar
PCA: Principal Component Analysis

## References

[1] Dubois B, Padovani A, Scheltens P, Rossi A, Dell’Agnello G (2016). Timely diagnosis for Alzheimer’s disease: a literature review on benefits and challenges. J Alzheimers Dis.

[2] Amjad H, Roth DL, Sheehan OC, Lyketsos CG, Wolff JL, Samus QM (2018). Underdiagnosis of dementia: an observational study of patterns in diagnosis and awareness in US older adults. J Gen Intern Med.

[3] Bradford A, Kunik ME, Schulz P, Williams SP, Singh H (2009). Missed and delayed diagnosis of dementia in primary care: prevalence and contributing factors. Alzheimer Dis Assoc Disord.

[4] Thyrian JR, Hoffmann W (2012). Dementia care and general physicians-a survey on prevalence, means, attitudes and recommendations. Cent Eur J Public Health.

[5] Ahmad S, Orrell M, Iliffe S, Gracie A (2010). GPs' attitudes, awareness, and practice regarding early diagnosis of dementia. Br J Gen Pract.

[6] Cahill S, Clark M, O'Connell H, Lawlor B, Coen RF, Walsh C (2008). The attitudes and practices of general practitioners regarding dementia diagnosis in Ireland. Int J Geriatr Psychiatry.

[7] Turner S, Iliffe S, Downs M, Wilcock J, Bryans M, Levin E, Keady J, O'Carroll R (2004). General practitioners' knowledge, confidence and attitudes in the diagnosis and management of dementia. Age Ageing.

[8] Perry M, Drašković I, Lucassen P, Vernooij-Dassen M, van Achterberg T, Rikkert MO (2011). Effects of educational interventions on primary dementia care: a systematic review. Int J Geriatr Psychiatry.

[9] Downs M, Turner S, Bryans M, Wilcock J, Keady J, Levin E, O'Carroll R, Howie K, Iliffe S (2006). Effectiveness of educational interventions in improving detection and management of dementia in primary care: cluster randomised controlled study. BMJ.

[10] Chodosh J, Berry E, Lee M, Connor K, DeMonte R, Ganiats T, Heikoff L, Rubenstein L, Mittman B, Vickrey B (2006). Effect of a dementia care management intervention on primary care provider knowledge, attitudes, and perceptions of quality of care. J Am Geriatr Soc.

[11] Kaduszkiewicz H, Bachmann C, van den Bussche H (2008). Telling "the truth" in dementia--do attitude and approach of general practitioners and specialists differ?. Patient Educ Couns.

[12] Moore V, Cahill S (2013). Diagnosis and disclosure of dementia–a comparative qualitative study of Irish and Swedish general practitioners. Aging Ment Health.

[13] O'Connor ML, McFadden SH. Development and Psychometric Validation of the Dementia Attitudes Scale. Int J Alzheimers Dis. 2010;2010:10. 10.4061/2010/454218.

[14] Liu JY-W, Lai C, Dai D, Ting S, Choi K (2013). Attitudes in the management of patients with dementia: comparison in doctors with and without special training. East Asian Arch Psychiatr.

[15] Mason RL, Annear MJ, Lo A, McInerney F, Tierney LT, Robinson AL (2016). Development and preliminary psychometric properties of the general practitioner attitudes and confidence scale (GPACS–D) for dementia. BMC Fam Pract.

[16] Bandura A (1998). Health promotion from the perspective of social cognitive theory. Psychol Health.

[17] Iliffe S, Manthorpe J, Eden A (2003). Sooner or later? Issues in the early diagnosis of dementia in general practice: a qualitative study. Fam Pract.

[18] Bamford C, Lamont S, Eccles M, Robinson L, May C, Bond J (2004). Disclosing a diagnosis of dementia: a systematic review. Int J Geriatr Psychiatry.

[19] Tabachnick BG, Fidell LS, Osterlind SJ: Using multivariate Statistics 2001.

[20] Adams KB, Matto HC, Sanders S (2004). Confirmatory factor analysis of the geriatric depression scale. The Gerontologist.

[21] Fang J, Power M, Lin Y, Zhang J, Hao Y, Chatterji S (2011). Development of short versions for the WHOQOL-OLD module. The Gerontologist.

[22] Bentler PM. EQS structural equations program manual. Encino. 1995:83–100.

[23] Hinkin TR, Tracey JB, Enz CA (1997). Scale construction: developing reliable and valid measurement instruments. J Hosp Tour Res.

[24] Schreiber JB, Nora A, Stage FK, Barlow EA, King J (2006). Reporting structural equation modeling and confirmatory factor analysis results: a review. J Educ Res.

[25] Holmes-Smith P (2000). Introduction to structural equation modelling using AMOS 4.0: course notes.

[26] Goodman A, Lamping DL, Ploubidis GB (2010). When to use broader internalising and externalising subscales instead of the hypothesised five subscales on the strengths and difficulties questionnaire (SDQ): data from British parents, teachers and children. J Abnorm Child Psychol.

[27] Floyd FJ, Widaman KF (1995). Factor analysis in the development and refinement of clinical assessment instruments. Psychol Assess.

[28] Maindal HT, Kayser L, Norgaard O, Bo A, Elsworth GR, Osborne RH (2016). Cultural adaptation and validation of the health literacy questionnaire (HLQ): robust nine-dimension Danish language confirmatory factor model. SpringerPlus.

[29] Jackson DL, Gillaspy JA, Purc-Stephenson R (2009). Reporting practices in confirmatory factor analysis: an overview and some recommendations. Psychol Methods.

[30] Guppy A, Edwards JA, Brough P, Peters-Bean KM, Sale C, Short E (2004). The psychometric properties of the short version of the cybernetic coping scale: a multigroup confirmatory factor analysis across four samples. J Occup Organ Psychol.

[31] Hooper D, Coughlan J, Mullen M. Structural equation modelling: Guidelines for determining model fit. Articles. 2008;2.

[32] Byrne BM. Structural equation modeling with Mplus: basic concepts, applications, and programming: Routledge; 2013.

[33] Graham JM (2006). Congeneric and (essentially) tau-equivalent estimates of score reliability: what they are and how to use them. Educ Psychol Meas.

[34] George JT, On behalf of the TDST, Warriner D, on behalf of the TDST, McGrane DJ, on behalf of the TDST, Rozario KS, on behalf of the TDST, Price HC, on behalf of the TDST et al: Lack of confidence among trainee doctors in the management of diabetes: the trainees own perception of delivery of care (TOPDOC) diabetes study. QJM: An Int J Med 2011, 104(9):761–766. 10.1093/qjmed/hcr046 PMC3158855 21511736

[35] Jacka D, Clode D, Patterson S, Wyman K (1999). Attitudes and practices of general practitioners training to work with drug-using patients. Drug Alcohol Rev.

[36] Vogt F, Hall S, Marteau TM (2005). General practitioners’ and family physicians’ negative beliefs and attitudes towards discussing smoking cessation with patients: a systematic review. Addiction.

[37] McAvoy B, Kaner E, Lock CA, Heather N, Gilvarry E (1999). Our healthier nation: are general practitioners willing and able to deliver? A survey of attitudes to and involvement in health promotion and lifestyle counselling. Br J Gen Pract.

[38] Ajzen I (1991). The theory of planned behavior. Organ Behav Hum Decis Process.

[39] Vernooij-Dassen MJ, Moniz-Cook ED, Woods RT, Lepeleire JD, Leuschner A, Zanetti O, Jd R, Kenny G, Franco M, Peters V (2005). Factors affecting timely recognition and diagnosis of dementia across Europe: from awareness to stigma. Int J Geriatr Psychiatry.

[40] Meuser TM, Boise L, Morris JC (2004). Clinical benefits and practices in dementia care: implications for health educators. Educ Gerontol.

[41] Foley T, Boyle S, Jennings A, Smithson WH (2017). **“**We’re certainly not in our comfort zone”: a qualitative study of GPs’ dementia-care educational needs. BMC Fam Pract.

[42] Tang EYH, Birdi R, Robinson L. Attitudes to diagnosis and management in dementia care: views of future general practitioners. Int Psychogeriatr. 2016:1–6. 10.1017/S1041610216001204 27502828

[43] Yaffe MJ, Orzeck P, Barylak L (2008). Family physicians’ perspectives on care of dementia patients and family caregivers. Can Fam Physician.

[44] Tsolaki M, Papaliagkas V, Anogianakis G, Bernabei R, Emre M, Frolich L, Visser PJ, Michel J-P, Pirttila T, Olde Rikkert M (2010). Consensus statement on dementia education and training in Europe. J Nutr Health Aging.

[45] Iliffe S, Wilcock J, Austin T, Walters K, Rait G, Turner S, Bryans M, Downs M (2002). Dementia diagnosis and management in primary care developing and testing educational models. Dementia.

[46] Aminzadeh F, Molnar FJ, Dalziel WB, Ayotte D (2012). A review of barriers and enablers to diagnosis and management of persons with dementia in primary care. Can Geriatr J.

